# The Influence of Various Role Models on Children’s Pro-environmental Behaviours

**DOI:** 10.3389/fpsyg.2022.873078

**Published:** 2022-05-20

**Authors:** Mingyue Liang, Qianying Chen, Yanyan Zhou

**Affiliations:** ^1^College of Teacher Education, Ningbo University, Ningbo, China; ^2^Department of Psychology, Ningbo University, Ningbo, China

**Keywords:** social learning theory, pro-environmental behaviours, teacher model, peer model, children

## Abstract

Although most schoolchildren can dispose of their own litter, they are typically not sensitive to environmental issues in the school’s public areas. How do we improve children’s sensitivity to public environments and cultivate pro-environmental behaviours? Based on Bandura’s social learning theory, this study explored the effects of various role models (teachers and peers) on the pro-environmental behaviours of children aged 7–13. A field study was conducted in which examples of postprandial garbage disposal behaviours were provided using role models and the subsequent behaviours of the children were observed. We located the experiment in a real educational context and manipulated the type of role model (teacher or peer) and the behaviour being modelled (positive behaviours involving picking up litter or negative behaviours involving littering). The results showed that different role models had different effects on the subjects’ pro-environmental behaviours. Only positive demonstration by teachers significantly improved the subjects’ pro-environmental behaviours, that is, teachers’ picking up of garbage in front of children significantly improved the children’s attention to the environment and their adoption of pro-environmental behaviours. Positive demonstration by peers, negative demonstration by teachers and negative demonstration by peers had no impact on the children’s pro-environmental behaviours. The results demonstrate that teachers must be mindful of their role as role models in the educational environment and facilitate students’ development of pro-environmental behaviours.

## Introduction

Pro-environmental behaviours are those that prioritise respect for the environment. They aim to protect the environment (Krajhanzl, 2010) and consciously reduce people’s negative impact on the natural and man-made world (Kollmuss and Agyeman, 2002). The majority of previous research on pro-environmental behaviour has focused on areas such as diet, consumption and water resources. For example, scholars have examined the reduction of food waste (Sorokowska et al., 2020) and the use of reusable bags by consumers (Isbanner et al., 2021) as pro-environmental behaviours. Other scholars have investigated children’s or young people’s understanding of attitudes towards oceans, lakes and the management of household water resources (Damerell et al., 2013; Votruba and Corman, 2020; Canosa et al., 2021). Although the environment is the cornerstone of human existence, many people do not exhibit either the awareness or the behaviours to protect it (Gifford and Nilsson, 2014), which directly affects the quality of human life (Hamzah, 2013). Therefore, the cultivation of pro-environmental behaviours among individuals in society, especially at a young age, is crucial (Kos et al., 2016; Chankrajang and Muttarak, 2017; Wu, 2018; Charry and Parguel, 2019; Palomo-Vélez et al., 2020; Pearce et al., 2021; Zeiske et al., 2021). Environmental education is the most effective tool available to raise awareness of future challenges regarding the environment and the ways to protect it (Heidari and Heidari, 2015). Consequently, a body of research has attempted to enhance children’s awareness through environmental education with the objective of cultivating pro-environmental behaviours (Collado et al., 2015; Collado and Corraliza, 2015; Otto and Pensini, 2017; Hoffmann and Muttarak, 2020; Torkar et al., 2020).

Environmental education mainly focuses on three aspects: active promotion of green schools to educate students on environmental protection (Wee et al., 2018; Kerret et al., 2020; Prasetiyo et al., 2020; Verma and Grover, 2021); integration of environmental protection education with chemistry (Osunji, 2021), geography (Aliman, 2019), science, technology, engineering and mathematics (STEM; Paulsen and Andrews, 2019; Ratnaningsih, 2020) and other disciplines, thereby infusing environmental protection education into subject education; and adoption of media such as videos and video games to popularise the value of environmental protection (Marlow, 2012; Fokides and Kefallinou, 2020; Safitri et al., 2021). Although previous attempts have achieved some success in raising awareness, it remains unclear whether they improve children’s actual pro-environmental behaviours. There is a significant discrepancy between environmental knowledge and awareness on the one hand, and observed environmental behaviours on the other hand (Jurin and Fortner, 2002). Indeed, most students fail to exhibit sufficient regard for the school environment, and outside of classrooms they are often seen littering the campus (Asmuni et al., 2012).

Many researchers have investigated the factors influencing children’s pro-environmental behaviours, such as environmental literacy (Wong et al., 2018), environmental policy (Cincera and Krajhanzl, 2013), environmental protection education (Cincera et al., 2012), pro-environmental cues and situational strength (Runhaar et al., 2019), task difficulty of pro-environmental behaviours (Runhaar et al., 2019), role models (i.e., teachers and staff; Higgs and McMillan, 2006), students’ gender, pro-environmental attitudes (PEA) and intentions (Runhaar et al., 2019), students’ ethical (pro-environmental) values, affective factors (neighbourhood attachment) and cognitive factors (perceived behavioural control; Rioux, 2011). These studies have mainly focused on children’s cognition of the environment along with individual differences. Although such a focus may lead to an increase in children’s environmental awareness, its effect on actual behaviour is not clear. Studies have shown that children’s pro-environmental behaviours still need to be improved. Despite the deepening environmental crisis, the proportion of children expressing concern about important environmental issues has decreased over the past 20 years (Ivanova, 2019).

A considerable number of studies have tested the effectiveness of specific interventions designed to improve children’s pro-environmental behaviour in relation to the above influencing factors. Five broad types of intervention have been studied. The first group of interventions consists of teaching activities such as painting (Bezzon da Silva and Soares da Silva, 2013; Vilá et al., 2020; Yeşilyurt et al., 2020), games (Loukia et al., 2021), using photos (Choi and Jo, 2012) and viewing plants (Choque and Teresa, 2021). The second consists of environmental education (EE) programmes developed by schools to improve students’ pro-environmental behaviour, such as hope-enhancing pro-environmental programmes (Kerret et al., 2020), residential outdoor environmental education programmes (Mullenbach et al., 2019) and the Children and Trees Growing Together programme (Dolins et al., 2010). The third group of interventions involves creating situations and pro-environmental cues to promote pro-environmental behaviours in students based on characteristics such as age (Corraliza et al., 2013) and pro-environmental attitudes and intentions (Fishbein and Ajzen, 1977). One such study examined three schools: School A tried to encourage pro-environmental behaviour by providing a hydration system, School B provided and promoted healthier food in the canteen and School C made furniture from recycled wood (Runhaar et al., 2019). In the fourth group of interventions, teacher role models are provided so that students can observe and learn pro-environmental behaviours such as turning off lights when one leaves the room and consuming organic local foods in minimal disposable packaging (Higgs and McMillan, 2006). This group of interventions includes those that train teachers to teach EE concepts and skills (Scott and Sulsberger, 2019; Varela-Losada et al., 2019). The fifth group of interventions aims to promote pro-environmental behaviours through various experiential outdoor activities, such as using vegetable gardens for environmental education and food re-education (Salles et al., 2020), visiting botanical gardens (Bissinger and Bogner, 2018) and zoo camp experiences or visits to aquariums (Collins et al., 2020).

The above studies have shown that interactive and experiential environmental education activities can effectively improve students’ pro-environmental behaviours. However, most of these studies involved specifically designed environmental education activities, either as individual observations of students or as group education. The research process in such studies is relatively complicated. For example, it is necessary to design an education plan that is appropriate to the development of all of the students at the school, as well as to take students outside the school to carry out outdoor environmental education activities. These external activities are costly in terms of paid staff hours, time and material resources. An alternative approach is to improve students’ pro-environmental behaviours *via* interventions that are quick, high-impact and low-cost (Popescu et al., 2020). Following this approach, this study investigated observational learning. We chose to observe a particular, easily observable pro-environmental behaviour for a fixed period after meals. This made the research process straightforward and practical, gave the researchers more time to observe the children’s behaviour and was less prone to risking deterioration in the environment due to the interactions and experiences involved. The observational learning approach is based on Bandura’s (1969, 1977) social (observational) learning theory. This theory aims to explain the process underlying changes in people’s behaviour and the factors influencing them. It posits that a learner acquires a specific behaviour from a model by observing the behaviour demonstrated by the model (Bandura, 1969, 1977). Bandura (1972) proposed that there are at least three reasons why role models influence children’s behaviour. First, by observing the behaviour of the model, children learn to behave in the same manner. Second, through the model, children understand the potential consequences of adopting a certain behaviour. Third, the model can inform children on how to behave in unfamiliar situations. Social learning theory has influenced many studies on behaviour change, such as that pertaining to health (Rosenstock et al., 1988), leadership (Sims and Manz, 1982), academic dishonesty (Hendy et al., 2021) and English language learning (Muir et al., 2021).

According to prior research, there are two key mechanisms through which role models influence learners. First, role models affect children’s thoughts, attitudes, values and comprehension abilities *via* direct methods of instruction, such as by improving classroom participation and communication skills in primary school students (Boyd et al., 2007), improving self-acceptance in students with learning disabilities (Guindon, 1993) and encouraging bystander intervention to prevent school bullying (Ioverno et al., 2021) and child violence (Chen et al., 2016). Second, children learn from role models by observing their actions and behaviours. Observational learning based on teachers or peers as role models can improve students’ academic performance in disciplines such as mathematics (Jung and Brady, 2016), English (Sadeghi and Sahragard, 2016) and clinical medicine (Potisek et al., 2019; Khan et al., 2020; Mohammadi et al., 2020, 2021). Studies on behaviour change guided by observational learning theory have primarily evaluated outcomes based on hypothetical situations. For example, research methods based on hypothetical situations have been widely used to examine students’ behavioural responses to conflict situations and social dilemmas (Johnson et al., 2001; Timler, 2008; Lozano, 2016). The drawback of this approach is that in hypothetical situations, subjects may be prone to enacting or reporting socially desirable behaviours that reflect ideal rather than actual behaviours. Moreover, these studies have not compared the roles of teachers and peer students as models, and it is not sufficient to base the design of targeted educational strategies solely on the behavioural characteristics of students.

To address this research gap, we conducted a field experiment to investigate the effects of different modelling behaviours on children’s pro-environmental behaviours in real situations relevant to their daily lives. Specifically, we examined the effects of various aspects of role modelling in teaching students how to deal with food waste on campus. We located the experiment in a real educational context and manipulated the type of role model (teacher or peer) and the behaviour being modelled (positive behaviours involving picking up litter or negative behaviours involving littering). Children aged 7–13 years were selected as subjects and were involved in the field study without their knowledge. Children in this age group belong to the compulsory education stage and were chosen as the participants in the study for two reasons. First, many scholars have shown that it is very important to cultivate children’s moral behaviour (including pro-environmental behaviour) at a young age (Talwar et al., 2016; Peplak and Malti, 2017; Leduc et al., 2018; Liberman et al., 2018; Hao and Wu, 2019). Second, the entire primary school stage is a critical period for developing good moral behaviour habits in children (Lin, 2018). Bandura’s observational learning theory suggests that a teacher’s role as a model is more prominent than that of student peers in a campus environment (Faulstich-Wieland, 2013; Muhamad et al., 2013; Tonga, 2014; Basheer et al., 2016; Wall and Hall, 2016; Sampermans and Claes, 2018; Cheung, 2020; Laguna et al., 2020; Moore et al., 2020). Therefore, we predicted that pro-environmental behaviours demonstrated by teachers would improve children’s pro-environmental behaviours. Because teachers play a positive social role by educating people, this uplifting effect may only occur when a teacher demonstrates positive litter-picking behaviour. However, the prosocial effects of peer modelling on children may be weak or even non-existent because children aged 7–13 are less dependent on peers (Lin, 2018). Nevertheless, we examined the possibility that behaviours modelled by peers also affect children, because studies have shown that children’s behaviours at this stage are influenced by peers to a certain extent (Carr et al., 2016; van Hoorn et al., 2016; Lease et al., 2020; Luo et al., 2020; Chung et al., 2021; Nenniger and Müller, 2021).

## Materials and Methods

### Subjects

We adopted a single-factor, five-level between-subjects experimental design. As suggested by Cohen (1988), the expected alpha value was set to 0.05, the statistical validity was set to 0.90 and the effect size was set to the medium level *f* = 0.25. Based on these criteria, the minimum sample size required was calculated using G*Power 3.0.10 and found to be 255. To satisfy this sample size requirement, 290 subjects were selected from a school in China offering the nine-year compulsory curriculum. The school was located in a city, and all subjects were from Zhejiang Province or nearby provinces and cities. To ensure the validity of the data, following the experiment, we asked all subjects whether they noticed the garbage on the ground and whether they noticed their teachers and peers dropping or picking up litter. If a subject did not notice these, they were removed from the sample. The final sample consisted of 285 subjects (144 boys and 141 girls) aged 7–13 years (*M* = 10.06, *SD* = 2.22). After the experiment, the experimenter explained the purpose of the experiment to the subjects and obtained permission from the children involved and their parents. The research was reviewed and approved by the Academic Committee of the College of Teacher Education, Ningbo University.

### Experimental Procedure and Design

#### Experimental Design

The study adopted a single-factor between-subjects design with the demonstration condition as the independent variable. Each subject was exposed to one of the following five conditions.

Control condition: As in all of the conditions, the researchers randomly threw more than 10 pairs of used disposable chopsticks near the trash can, implying to the subjects that ‘everyone is throwing chopsticks here’. Fifty-eight subjects (29 boys and 29 girls) were assigned to this condition.

Negative demonstration by teacher model (NDTM): The researchers invited a teacher from the school to serve as a role model in this condition. When the teacher saw the subjects approaching the trash can, he said, ‘It’s dirty here’, and in their presence, carelessly tossed his chopsticks into the pile on the floor around the trash can. Fifty-eight subjects (29 boys and 29 girls) were assigned to this condition.

Positive demonstration by teacher model (PDTM): The researchers invited another teacher from the school to serve as a role model in this condition. When the teacher saw the subjects approaching the trash can, he said, ‘It’s dirty here’, and in their presence, picked up several chopsticks from the floor and threw them into the trash can. Fifty-eight subjects (29 boys and 29 girls) were assigned to this condition.

Negative demonstration by peer model (NDPM): The researchers invited a student from the school to serve as a role model in this condition. When the student saw the subjects approaching the trash can, he said, ‘It’s dirty here’, and in their presence, carelessly tossed his chopsticks into the pile on the floor around the trash can. Fifty-five subjects (28 boys and 27 girls) were assigned to this condition.

Positive demonstration by peer model (PDPM): The researchers invited another student from the school to serve as a role model in this condition. When the student saw the subjects approaching the trash can, he said ‘It’s dirty here’, and in their presence, picked up several chopsticks from the floor and threw them into the trash can. Fifty-six subjects (29 boys and 27 girls) were assigned to this condition.

#### Scenario Design and Experimental Process

**Figure 1:**
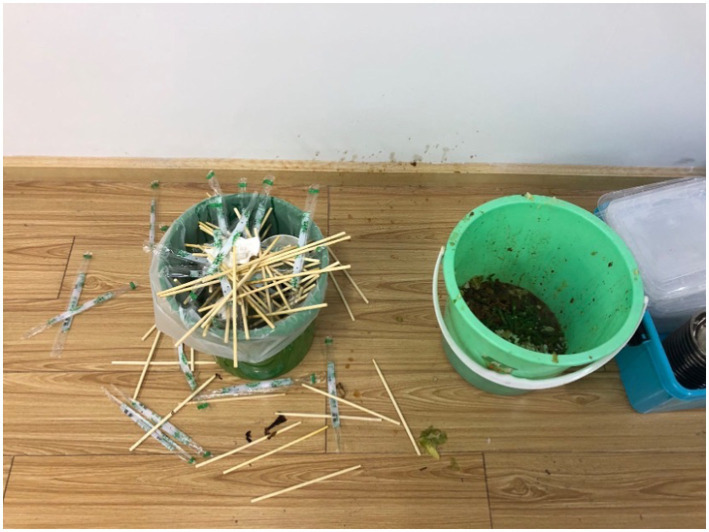
The designated garbage disposal area.

We adopted the field experiment method in this study. The specific experimental situation was as follows. At around 11.30 am every day during the course of the experiment, with the cooperation of the teacher, the experimenter delivered lunches packed in take-out boxes to the classroom. Each lunch included a pair of disposable chopsticks. The subjects were asked to eat their lunches in their respective classrooms. The teacher informed the subjects of the designated place to dispose of their food waste (including the disposable chopsticks) after the meal, which was located in a public area in the corridor outside the classroom. After the meal, each subject went separately to the designated area to dispose of their food waste. There were three trash cans in the designated area, one each for chopsticks, leftovers and take-out boxes. The immediate area around the trash can for chopsticks was arranged haphazardly, which provided the subjects with the environmental cue that ‘everyone throws chopsticks here’. In this study, disposable chopsticks were used as observation items for two reasons. First, eating with chopsticks is a standard Chinese dining habit, and the school typically issues a pair of disposable chopsticks to each student due to hygiene considerations, making them easily available to the subjects. Second, disposable chopsticks are easy to observe and quantify, while leftovers and take-out boxes are not easily observable or quantifiable. If the area had been altered by a subject as they disposed of their food waste, the area was rearranged to its initial state after they had returned to the classroom and before the next subject arrived. Figure 1 shows the initial state of the area.

Based on the work of Reno et al. (1993), the subjects’ behaviours were coded into three categories: pick-up, throw-down and walk-by. Reno et al. (1993) used a handbill that was placed under the windshield wiper on the side of the car, observing whether the driver littered using the flyer. They defined littering as the act of ‘throwing the handbill in the environment outside the car’ (no trash can was provided in the experimental scenario). Consistent with this definition, during the experiments in the current study, the experimenter recorded the subjects’ behaviours under different demonstration conditions from a hidden location. Options included picking up chopsticks from the ground and putting them in the trash can, throwing their chopsticks on the ground, or just putting their own chopsticks in the trash can without touching the others.

As mentioned, all of the subjects in the experimental conditions were asked whether they noticed the model (teacher or peer) picking up or throwing litter in front of the trash can, and only those who did notice this were included in the final sample. Furthermore, we verified that none of the subjects were aware that they were involved in an experiment, and therefore, the results were not affected by any potential attempts to conform to the expectations of the experiment.

## Pre-processing

In this study, we defined ‘pick-up’ behaviour as follows: subjects noticed the garbage on the ground outside the trash can, threw their used take-out boxes, chopsticks and leftovers into the trash can and then bent down to pick up the chopsticks on the ground outside the trash can and put them inside. We defined ‘throw-down’ as follows: subjects noticed the garbage on the ground, threw their used take-out boxes and leftovers into the bin and threw their used chopsticks in a place other than the trash can. We defined ‘walk-by’ as follows: subjects noticed the garbage on the ground outside the garbage can and threw their used chopsticks, take-out boxes and leftovers inside without picking up the chopsticks on the ground.

There were two test indicators to determine how the subjects noticed the garbage on the ground outside the trash can. First, a confederate made the observation ‘It’s dirty here’ to remind subjects to notice the garbage on the ground. Second, after the experiment, the experimental video was watched to confirm that subjects’ eyes had moved to the garbage on the ground. We separately invited two experts who were not aware of the purpose of the experiment to watch the video recordings and code the behaviours of the subjects. We allocated the values of 0, 1 and 2 to pick-up, throw-down and walk-by, respectively, and the experts’ coding was later evaluated for consistency (see Table 1).

**Table 1:** Crosstabulation of coding consistency check.

Demonstration conditions		Frequency (Percentage)		
		Pick-up	Throw-down	Walk-by
Control (*n* = 58)	Rater1	2 (3.45%)	0 (0.00%)	56 (96.55%)
	Rater2	2 (3.45%)	0 (0.00%)	56 (96.55%)
Negative demonstration by teacher (*n* = 58)	Rater1	2 (3.45%)	1 (1.72%)	55 (94.83%)
	Rater2	2 (3.45%)	1 (1.72%)	55 (94.83%)
Positive demonstration by teacher (*n* = 58)	Rater1	13 (22.41%)	1 (1.72%)	44 (75.86%)
	Rater2	14 (24.14%)	1 (1.72%)	43 (74.14%)
Negative demonstration by peer (*n* = 55)	Rater1	1 (1.82%)	1 (1.82%)	53 (96.36%)
	Rater2	1 (1.82%)	0 (0.00%)	54 (98.18%)
Positive demonstration by peer (*n* = 56)	Rater1	5 (8.93%)	0 (0.00%)	51 (91.07%)
	Rater2	3 (5.36%)	0 (0.00%)	53 (94.64%)

In total, there were 285 valid cases with no missing data (*N* = 285). Cronbach’s alpha coefficient was 0.994, indicating a high level of inter-rater reliability.

## Results

To examine the influence of the behaviours demonstrated by the models on the subjects’ behaviours, we compared the results from the four experimental conditions with those of the control condition by using the abovementioned three behaviours. Due to the pre-processing of the data, the data we obtained were frequency data. For this reason, our data analysis was conducted using nonparametric tests.

### Effect of Negative Demonstrations by Models

**Figure 2:**
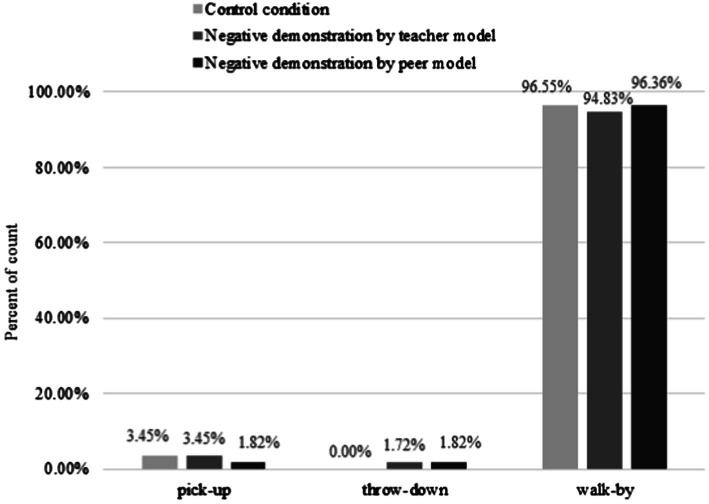
Percentage of subjects engaging in the three target behaviours in the Control, NDTM, and NDPM conditions.

First, Fisher’s exact test was used to check for differences between the NDTM and control group subjects in terms of their adoption of the three behaviours. The results are illustrated in Figure 2. There was no significant association between these two conditions and the subjects’ adoption of the three behaviours ( x *^2^*_(2, 116)_ = 1.608, *p* = 1.000). This implies that there was no significant difference in the proportion of subjects picking up (NDTM: 3.45% vs. control: 3.45%), throwing down (NDTM: 1.72% vs. control: 0%) or walking by (NDTM: 94.83% vs. control: 96.55%) between the two groups, indicating that the teacher’s negative demonstration did not affect the subjects’ pro-environmental behaviours.

Correspondingly, we also compared the differences in the subjects’ behaviours between the NDPM and control conditions (see Figure 2). There was no significant correlation between these two conditions and the subjects’ adoption of the behaviours ( x *^2^*_(2, 113)_ = 1.351, *p* = 0.802). Specifically, there was no significant difference in the proportion of subjects picking up (NDPM: 1.82% vs. control: 3.45%), throwing down (NDPM: 1.82% vs. control: 0%) or walking by (NDPM: 96.36% vs. control: 96.55%) between the two groups, indicating that the peer’s negative demonstration did not affect the pro-environmental behaviours of the subjects.

The above results show that negative demonstrations by the models neither affected the subjects’ pro-environmental behaviours nor promoted behaviours detrimental to the environment.

### Effect of Positive Demonstrations by Models

**Figure 3:**
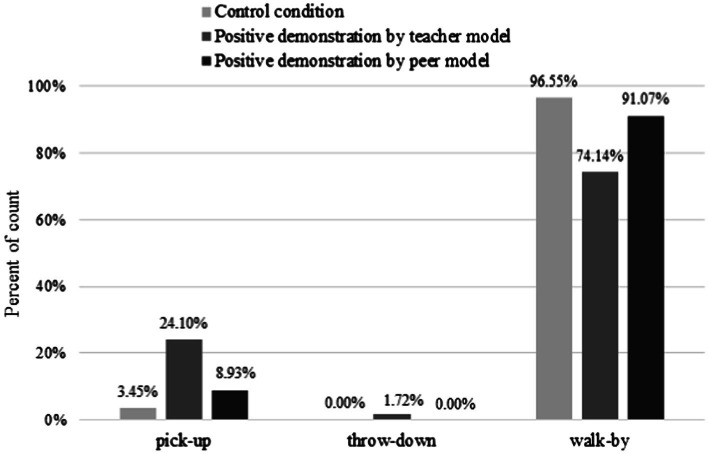
Percentage of subjects engaging the three target behaviours in the Control, PDTM, and PDPM conditions.

To examine the differences between the PDTM and control group subjects in terms of their adoption of pro-environmental behaviours, we used Fisher’s exact test to compare the proportion of subjects that adopted the three behaviours between the two conditions. These results are illustrated in Figure 3. There was a significant association between the conditions and the type of behaviour adopted by the subjects ( x *^2^*_(2, 116)_ = 12.012, *p* < 0.001). Specifically, there was a difference in the proportion of subjects picking up (PDTM: 24.14% vs. control: 3.45%), throwing down (PDTM: 1.72% vs. control: 0%) or walking by (PDTM: 74.14% vs. control: 96.55%) between the two groups, indicating that compared with the control condition, the teacher’s positive demonstration affected the subjects’ pro-environmental behaviours.

To further analyse this effect, a post-hoc test was used. Based on the standard suggested by Agresti (2002, p. 81), if the absolute value of the adjusted standardised residual was greater than 2, we considered the difference between the observed frequency and the expected frequency to be statistically significant. The results are summarised in Table 2. The absolute value of the adjusted standardised residual for the pick-up and walk-by behaviours was 3.2 and 3.4, respectively. This shows that teachers’ positive demonstration can significantly improve children’s pro-environmental behaviour of picking up litter and inhibit the environmentally neglectful behaviour of walking by, but it may not change children’s environmentally damaging behaviour of throwing litter.

**Table 2:** Crosstabulation of model’s behaviour type and subject’s behaviour type.

Type of model behaviour	Type of subject behaviour		
	Pick-up	Thrown-down	Walk-by
Control	2 (3.2)	0 (−1.0)	56 (3.4)
Positive Demonstration by Teacher Model	14 (3.2)	1(1.0)	43 (−3.4)

Adjusted residuals appear in parentheses below observed frequencies.

Similarly, to verify whether positive demonstration by the peer affected the subjects’ adoption of the three behaviours, we compared the PDPM and control group subjects’ adoption of the three behaviours (see Figure 3). There was no significant difference in the proportional distribution of the subjects’ adoption of the three behaviours between the two groups ( x *^2^*_(1, 114)_ = 0.686, *p* = 0.407). This implies that there was no difference between the two groups in the proportion of subjects picking up (PDPM: 8.93% vs. control: 3.45%), throwing down (PDPM: 0% vs. control: 0%) or walking by (PDPM: 91.07% vs. control: 96.55%), indicating that positive demonstration by the peer did not affect the subjects’ pro-environmental behaviours.

The above results show that the teacher’s positive demonstration increased the subjects’ adoption of pro-environmental behaviours and reduced their indifference to the environment (walk-by), but it did not affect their litter-throwing behaviour. However, positive demonstration by the peer did not have a significant impact on the subjects’ pro-environmental behaviours.

### Effect of Model Type

To check for differences in the effects of the same behaviours when demonstrated by different models, we compared the effects of demonstrations by teachers and peers on the subjects’ behaviours under the positive and negative demonstration conditions.

The distributions of the number of subjects in the NDTM and NDPM groups for the three behaviours were not significantly different ( x *^2^*_(2,113)_ = 0.612, *p* = 1.000). This implies that there was no difference in the proportion of subjects in the two groups picking up (NDTM: 3.45% vs. NDPM: 1.82%), throwing down (NDTM: 1.72% vs. NDPM: 1.82%) or walking by (NDTM: 94.83% vs. NDPM: 96.36%). This shows that in a messy environment, irrespective of whether the model is a teacher or a peer, demonstrations of negative behaviours do not affect the subjects’ pro-environmental behaviours.

However, the distributions of the number of subjects in the PDTM and PDPM groups for the three behaviours were significantly different ( x *^2^*_(2, 114)_ = 5.797, *p* = 0.033). A post-hoc test (see Table 3) showed that the absolute value of the adjusted standardised residual for the pick-up behaviour was 2.2, and the absolute value of the adjusted standardised residual for the walk-by behaviour was 2.4. This shows that positive demonstration by the teacher was significantly more effective than that by the peer in improving children’s pro-environmental behaviour of picking up litter and in inhibiting the environmentally neglectful behaviour of walking by. However, there was no significant difference in the effect of these two models on children’s environmentally damaging behaviour of throwing litter.

**Table 3:** Crosstabulation of model behaviour type and subject behaviour type.

Type of model behaviour	Type of subject behaviour		
	Pick-up	Throw-down	Walk-by
Positive Demonstration by Teacher Model	14 (2.2)	1 (1.0)	43 (−2.4)
Positive Demonstration by Peer Model	5 (−2.2)	0 (−1.0)	51 (2.4)

Adjusted residuals appear in parentheses alongside the observed frequencies.

## Discussion

The purpose of this study was to investigate the effects of different role models on children’s pro-environmental behaviours in a real school environment. Therefore, we adopted a field experiment methodology based on social learning theory to examine how children dispose of their waste after meals, thus examining pro-environmental behaviours in a school environment with which children are very familiar. The results showed that (1) negative demonstrations by teachers and peers did not significantly increase instances of littering behaviour among the subjects; (2) positive demonstrations by teachers increased instances of litter-picking behaviour and reduced instances of walking by behaviour among the subjects but did not affect their garbage-throwing behaviour; and (3) compared with positive demonstrations by peers, those by teachers significantly increased instances of litter-picking behaviour and reduced instances of walking by behaviour among the subjects.

Studies on children’s environmental protection behaviours have primarily relied on questionnaires (Rioux, 2011; Cavaliere et al., 2018; Wang et al., 2021) and interviews (Schneller et al., 2015; Kos et al., 2016; Simonová and Cincera, 2016; Yeşilyurt al., 2020), and have examined the impact of environmental education on their awareness of environmental protection. They have however not satisfactorily explored whether this impact on awareness or knowledge is reflected in children’s behaviours. Our study addressed this gap by examining how children’s pro-environmental behaviours can actually be influenced. Situating the experiment in the children’s campus not only improved the ecological validity of the research, but also gave students a real sense of the importance of protecting their environment. In this study, we used disposable chopsticks as an experimental observation item. This is consistent with a similar study by Loschelder et al. (2019), which used a field experiment research method using disposable takeaway cups as an observation item to examine the impact of dynamic social norms on sustainable consumption by customers.

Consistent with the research hypothesis, teachers’ positive demonstrations promoted the subjects’ pro-environmental behaviours. This indicates that teachers have a considerable influence on children’s pro-environmental behaviours. Teachers are role models for their students, and their positive behaviours significantly influence their students. Similar results have been obtained in studies in other educational settings regarding the effects of positive behavioural demonstrations on other student behaviours. For example, Lazarowitz and Naim (2013) showed that relative to simple expository learning, teacher modelling of three-dimensional cellular models had a positive impact on students’ academic performance. Basheer et al. (2016) found that teacher demonstrations of redox reactions and electrolysis experiments had a positive impact on students’ understanding and mastery of experimental skills. In addition to academic performance, many scholars have focused on healthy student behaviours. For example, Cheung (2020) measured the number of steps pre-schoolers were taking using pedometers to measure their level of physical activity. The results showed that children had higher levels of physical activity in physical education classes taught by more active teachers than those in classes taught by less active teachers. The findings of Sisson et al. (2017) suggest that when childcare teachers serve as role models for healthy behaviours, this may be beneficial to the health of both children and teachers. This was also confirmed by Leman et al. (2021) for a much older age group: these researchers found that when medical teachers served as healthy role models in medical school, students’ healthy behaviours improved. Taken together, the above studies and the present study suggest that when teachers serve as role models for positive behaviours, this has a positive impact on the behaviour of their students. Teachers should therefore be conscious of their responsibilities as role models in educational settings.

However, positive demonstration by a peer did not significantly affect the children’s pro-environmental behaviours. These results show that teachers are fairly congruent with the conception of role models in children’s minds, while their peers are less so, and at least not as compatible as the peers that adults choose. This is consistent with Bandura’s argument that a prerequisite for an individual to be a role model in the education of a subject is the subject’s acceptance of and agreement with the role model and their behaviours (Huang and Han, 2008). The results of this study suggest that teachers are role models that children identify with, but the choice of peers as role models to influence children’s behaviours in studies and interventions may need to be reconsidered. Although peer role models did not exert a significant influence in this study, some studies have indeed shown that peer role models affect children’s behaviour. For example, Gardner and Steinberg (2005) found that adolescents were more likely to be negatively influenced by their peers than adults, while Ivaniushina and Titkova (2021) found that adolescents adjusted their drinking behaviours to accommodate the behaviour of their peers. It is therefore possible that the influence of peer role models may be different for different behaviours. For this reason, in educational practice, it is necessary to choose individuals who are likely to be accepted and recognised by students and who demonstrate positive behaviours as role models, as this may contribute more effectively to encouraging pro-environmental behaviours.

In contrast to the significant effects of positive demonstrations on children’s pro-environmental behaviours, negative demonstrations by teachers or peers did not affect the children’s litter-throwing behaviour. Other studies have also confirmed this. For example, Hegarty et al. (2020) examined the effects of teacher and parent behaviours on children’s behaviours and found that the sedentary behaviours of teachers and parents had little effect on the sedentary behaviours of children. In our study, the children had probably acquired relevant knowledge and behaviours on the protection of the environment during their schooling. Consequently, they had a certain understanding of and adherence to the norms, enabling them to avoid blindly imitating their teachers’ questionable behaviours. Bandura (1977) found that momentary external influences do not change children’s behaviours. During their adoption of pro-environmental behaviours, children’s personal norms are activated. The activation of personal norms requires two conditions. The first is that the individual recognises the adverse consequences that will be caused to others by not performing prosocial behaviours, that is, the cognition of the result. The second is that the individual believes that they are responsible for these adverse consequences, that is, the attribution of responsibility (Schwartz, 1977). Children’s pro-environmental behaviours result in external evaluations from teachers, peers and other key individuals in their lives, and these in turn facilitate their transformation into self-sustaining behaviours, causing the internalisation of external social norms as personal norms. Moreover, students have a certain level of independent thinking in which rationality and self-regulation play a role. It is important, however, to emphasise that there is still a risk that negative behavioural modelling by teachers may negatively affect students’ behaviour. Masoumpoor et al. (2017), for example showed that uncivilised behaviour from teachers in nursing education is destructive to the teaching and to the students’ learning environment. It is also important that modelling be done in the right way, as any behaviour that causes humiliation and embarrassment to students may have an effect opposite to that intended.

In the baseline condition of this study, a messy environment was prepared, providing children with an environmental cue that ‘everyone is littering here’. A key motivation for adopting this arrangement was that despite advancements in human society, many children still grow up in difficult circumstances. For example, for children from disadvantaged backgrounds, the school lunch may be the most important meal of the day (Colquhoun et al., 2008). Therefore, understanding the factors that influence children’s consumption in the school meal environment is crucial to changing their eating habits (South et al., 2012). Children from families with low socio-economic status and those whose parents have low education levels are at risk of developmental delays, and it is therefore necessary to conduct activities that allow these children to access good healthcare, nutrition and dietary habits (Buonomo et al., 2020). The purpose of this research was to support children’s learning and awareness in messy environments: even if the situation is difficult, schools and teachers can still develop pro-environmental habits and qualities in children through the power of education.

Finally, although this study provides valuable insights, there are certain limitations. First, the subjects in this study were children in the compulsory education stage, so it is unclear whether the results of this study can be extrapolated to other age groups. In future research, it would be fruitful to use the same research design. Second, in this study, the behaviours demonstrated by peers had little effect on the children, which may be because the selected peers were not accepted and recognised by the subjects as role models. In future studies, influential peers could be specifically selected to investigate whether they affect children’s adoption of positive behaviours. Third, the results of this study showed that demonstrations of positive behaviour by teachers can promote pro-environmental behaviours in children, but it is uncertain whether these positive effects can be extrapolated to other behaviours, such as helping behaviour or leadership behaviour. In future research, we may examine other behaviours to determine whether the type of role model used has a differential effect. Fourth, previous studies have shown that family socio-economic status affects children’s behaviour (Jansen et al., 2012; Tandon et al., 2012), but this study did not collect this information. The potential influence of this factor could be included in future research. Despite these limitations, we believe that the results of this study provide new information about the importance of teacher role models in educational settings for students, including the fact that often students do not simply imitate their teachers but instead demonstrate independent thinking in determining their own behaviour.

## Data Availability Statement

The raw data supporting the conclusions of this article will be made available by the authors, without undue reservation.

## Ethics Statement

The studies involving human participants were reviewed and approved by Ningbo University Ethics Committee. Written informed consent to participate in this study was provided by the participants’ legal guardian/next of kin.

## Author Contributions

All authors listed have made a substantial, direct, and intellectual contribution to the work and approved it for publication.

## Funding

This study was funded by the 2019 General Project of Education “Research on the Mechanism of Cultivating Adolescent Values from the Perspective of Nudge Theory” (no. BEA190111) of the National Social Science Foundation of China.

## Conflict of Interest

The authors declare that the research was conducted in the absence of any commercial or financial relationships that could be construed as a potential conflict of interest.

## Publisher’s Note

All claims expressed in this article are solely those of the authors and do not necessarily represent those of their affiliated organizations, or those of the publisher, the editors and the reviewers. Any product that may be evaluated in this article, or claim that may be made by its manufacturer, is not guaranteed or endorsed by the publisher.
